# Multilocus Genetic Variants in a Child With Neuro‐Ichthyosis: A Case of Pharmacoresistant Epilepsy and Developmental Delay Associated With CC2D2A, ABCA12, DOCK6 Variants, and a 14q31.3–q32.11 Deletion

**DOI:** 10.1002/ccr3.71861

**Published:** 2026-01-14

**Authors:** Bessan Hamed Dababseh, Ala'a S. Ghnimat, Lubna W. AbuHamdiya, Mones H. Atatre, Ahmad Batran, Mahdi W. Suboh

**Affiliations:** ^1^ Department of Clinical Medical Sciences, Faculty of Medicine and Health Sciences Palestine Polytechnic University Hebron Palestine; ^2^ Department of Internal Medicine Alia Governmental Hospital Hebron Palestine; ^3^ Faculty of Medicine and Health Sciences Palestine Polytechnic University Hebron Palestine

**Keywords:** CC2D2A, ichthyosis, Joubert syndrome, neuro‐ichthyosis, pharmacoresistant epilepsy

## Abstract

Neuro‐ichthyosis is a rare group of disorders characterized by the coexistence of neurological dysfunction and ichthyotic skin changes. We report a 5‐year‐old girl born to consanguineous parents who presented with pharmacoresistant epilepsy, severe developmental delay, microcephaly, and ichthyosis. Family history revealed similarly affected siblings, suggesting a hereditary basis. Despite multiple antiepileptic drugs, seizures remained uncontrolled. Whole exome sequencing identified a homozygous CC2D2A variant consistent with Joubert syndrome type 9, along with heterozygous variants in ABCA12 and DOCK6, and a 14q31.3–q32.11 deletion. This unique combination represents an unprecedented multilocus pathogenic mechanism contributing to the complex neurocutaneous phenotype. The findings expand the known genotypic and phenotypic spectrum of neuro‐ichthyosis and highlight the importance of early whole exome sequencing for accurate diagnosis, genetic counseling, and management of patients with severe neurocutaneous disorders, especially in consanguineous populations where multilocus pathogenicity may underlie atypical or severe presentations.

## Key Clinical Message

Early Whole Exome Sequencing is crucial for diagnosing complex neurocutaneous phenotypes, especially in consanguineous populations. This case highlights an unprecedented multilocus pathogenic mechanism involving CC2D2A (primary), ABCA12, DOCK6 variants, and a 14q31.3–q32.11 deletion (modifiers), which collectively shape the severe neuro‐ichthyosis phenotype and guide prognosis and management.

## 1 Introduction

Neuro‐ichthyosis represents a rare and heterogeneous group of disorders characterized by the coexistence of neurological dysfunction and ichthyotic skin abnormalities. These syndromes are exceedingly uncommon, with only a limited number of documented cases worldwide, and their true incidence remains unknown due to diagnostic complexity and phenotypic variability [1]. Most cases result from autosomal recessive inheritance, particularly in consanguineous populations, and involve disruptions in diverse biological pathways including vesicle trafficking, lipid metabolism, and ciliary function [1, 2]. The phenotypic presentation often includes global developmental delay, epilepsy, spasticity, intellectual disability, and severe ichthyosis, typically manifesting early in life and progressing over time [2, 3].

Advances in next‐generation sequencing, particularly whole exome sequencing (WES), have significantly improved the early diagnosis of neuro‐ichthyotic syndromes by identifying multilocus pathogenic variants that would otherwise go undetected [4, 5]. Genetic mutations in genes such as CC2D2A, ABCA12, and DOCK6 have been independently implicated in syndromic forms of Joubert syndrome, autosomal recessive congenital ichthyosis (ARCI), and Adams‐Oliver syndrome, respectively [1, 5, 6]. However, the concurrent presence of multiple pathogenic or likely pathogenic variants, as observed in our case, is exceptionally rare and highlights the need for a broader diagnostic perspective. Treatment is largely supportive and symptomatic, focusing on seizure control, skin care, and developmental therapies [3, 7]. Prognosis remains poor in cases with extensive multisystem involvement, especially when seizures are pharmacoresistant and cognitive and motor impairments are severe [7, 8].

Here, we report a unique case of a child with neuro‐ichthyosis presenting with pharmacoresistant epilepsy, severe developmental delay, and ichthyosis, in whom whole exome sequencing revealed multilocus pathogenic variants including CC2D2A, ABCA12, DOCK6, and a 14q31.3–q32.11 deletion. This constellation of genetic findings has not been previously described in a single patient and emphasizes the value of early genetic testing in complex neurocutaneous syndromes.

## 2 Case History/Examination

A 5‐year‐old girl with consanguineous parents presented with a syndromic neurocutaneous syndrome of pharmacoresistant seizures, developmental regression, and intractable xerosis from infancy. She was born following an uneventful full‐term pregnancy with normal birth weight and without perinatal complications. The clinical characteristics and genetic findings are summarized in Table 1.

**TABLE 1:** Summary of the chronological clinical course and key diagnostic interventions in the reported case of multilocus neuro‐ichthyosis.

Date/Age	Event/Clinical finding	Intervention/Investigation	Outcome/Notes
Infancy (Birth–4 months)	Normal pregnancy and delivery. Early infancy marked by hypotonia and poor head control by 4 months	Routine pediatric follow‐up	Developmental delay first noted
11 months	Delayed sitting; hypotonia persists	Neurological assessment initiated	Gross motor delay confirmed
13 months	First generalized tonic–clonic seizure	Hospital admission; EEG performed showing multifocal epileptiform discharges (frontal‐central). Started on antiepileptic medications (clobazam, levetiracetam, valproate)	Recurrent seizures; poor response to therapy
Infancy–3 years	Progressive developmental regression; no speech development; loss of social interaction by age 3	Serial neurological evaluations and developmental assessments	Severe global developmental delay documented
Early childhood (2–4 years)	Dry scaly skin on trunk and extremities; hyperlinearity of palms; mild plantar keratoderma	Dermatological evaluation	Ichthyosis diagnosed. Supportive skin care initiated
Age 3–4 years	Recurrent hospitalizations for status epilepticus	Escalation of antiepileptic regimen; seizure control attempts	Pharmacoresistant epilepsy established
Age 4–5 years	Persistent microcephaly, hypotonia with peripheral hypertonia, poor balance and vision tracking	MRI brain: high signal near posterior horns, no midline shift or hemorrhage	Imaging compatible with neurodevelopmental disorder; no acute pathology
Age 5 years	Suspected syndromic neurocutaneous disorder given combined neurological and dermatological findings	Whole Exome Sequencing (WES) performed	Identified multilocus pathogenic variants: CC2D2A (homozygous), ABCA12 (heterozygous VUS), DOCK6 (heterozygous likely pathogenic), and 14q31.3–q32.11 deletion
Post‐WES (age 5 years)	Genetic counseling for family; discussed prognosis and recurrence risk	Multidisciplinary management (neurology, dermatology, genetics)	Final diagnosis: multilocus neuro‐ichthyosis with poor prognosis
Current/Follow‐up	Persistent drug‐resistant seizures and severe developmental impairment	Ongoing supportive care: seizure management, physical and speech therapy, skin care	Prognosis remains poor; requires lifelong assistance and monitoring

The family history was notable for a brother with seizures and global developmental delay, as well as two other brothers who died in early infancy with ichthyotic skin changes. The parents are first‐degree cousins.

On physical examination, the patient had dry scaly skin on the trunk and extremities, the most marked on the extensor surfaces. The scales were fine to coarse in texture, adherent, and non‐inflammatory. Hyperlinearity of the palms and mild plantar keratoderma were observed. Neurological examination was indicative of microcephaly, axial hypotonia with peripheral hypertonia, brisk deep tendon reflexes, and poor visual tracking, though fundoscopy was unremarkable.

Concerns with development were apparent during the first year of life. She never developed expressive speech, was unable to control her head at 4 months, and demonstrated delayed sitting at 11 months. By age 3 years, she exhibited loss of social interaction and increasing aggressive conduct. During infancy, microcephaly was observed, and it continued until childhood. Hypotonia and poor balance caused a worldwide delay in motor development. She was still unable to walk on her own and was non‐verbal.

At 13 months of age, the patient had her first seizure. Over time, the generalized tonic–clonic seizures became more common, leading to repeated hospitalizations and recurring episodes of status epilepticus.

## 3 Methods (Differential Diagnosis, Investigations, and Treatment)

### 3.1 Investigations

Multifocal epileptiform discharges with a frontal‐central preponderance were seen on EEG. Several antiepileptic medications, such as clobazam, levetiracetam, and valproate did not produce long‐lasting seizure control.

Brain MRI showed high signal intensity adjacent to the posterior horns of the lateral ventricles. Crucially, the brainstem and cerebellum appeared normal, with no evidence of the classic Molar Tooth Sign (MTS) or hindbrain malformations typically associated with classic Joubert syndrome.

### 3.2 Whole Exome Sequencing Methodology

WES was performed using the Illumina NextSeq 500 platform with an average target coverage of > 100×. Reads were aligned to the human reference genome (GRCh37/hg19). Bioinformatic analysis utilized the GATK Best Practices pipeline for variant calling and annotation. Variants were filtered against the GnomAD database (population frequencies) and classified according to the ACMG/AMP guidelines as Pathogenic (P), Likely Pathogenic (LP), or Variant of Uncertain Significance (VUS).

Whole exome sequencing (WES) identified several key genetic findings. The main diagnosis is supported by a homozygous missense variant in the CC2D2A gene (c.1268G>A, p.Arg423Gln) which is associated with Joubert syndrome type 9 (JBTS9). This variant was confirmed by Sanger sequencing.

Additionally, a heterozygous variant of uncertain significance (VUS) was found in the ABCA12 gene (c.5779‐1G>A). This variant was confirmed by Sanger sequencing. This gene is associated with autosomal recessive congenital ichthyosis (ARCI), and the patient's family has a significant history of ichthyosis, with two deceased siblings exhibiting the condition. A heterozygous likely pathogenic variant was also detected in the DOCK6 gene (c.2520dupT), which is linked to Adams‐Oliver syndrome type 2 (AOS2). This variant was confirmed by Sanger sequencing.

Finally, a 1 Mbp heterozygous interstitial deletion in the 14q31.3–q32.11 region (Chr14: 93,892,109–94,892,109) was identified by WES and subsequently confirmed by Chromosomal Microarray (CMA). The deletion is classified as likely pathogenic and encompasses several key neurodevelopmental genes, including FOXG1, BCL11B, SERPINA1, SCGN, and MIPOL.

Based on molecular and imaging evidence, the patient satisfies the criteria for neuroichthyosis because of the co‐occurrence of ichthyosis, global developmental delay, cerebellar malformation, and drug‐resistant epilepsy. The main diagnosis is CC2D2A‐related Joubert syndrome, with the severity of the neurological and cutaneous symptoms being influenced by other locus modifying variables.

Given the severity of the multisystem involvement, seizure burden, and neurodevelopmental impairment, the prognosis is poor. Lifelong reliance, severe cognitive impairment, and an ongoing risk of refractory seizures and recurring infections are among the long‐term consequences.

## 4 Treatment

Treatment is largely supportive and symptomatic, focusing on seizure control, skin care, and developmental therapies [3, 7].

## 5 Conclusion and Result (Outcome and Follow Up)

This case report details a complex multilocus presentation of neuro‐ichthyosis in a patient with pharmacoresistant epilepsy, severe developmental delay, and ichthyosis. This severe phenotype is attributed to a complex genetic background, where the CC2D2A‐related Joubert syndrome type 9 (JBTS9) is the primary diagnosis, and the heterozygous variants in ABCA12 and DOCK6, along with the 14q31.3–q32.11 deletion, act as likely genetic modifiers.

The findings highlight the importance of early whole exome sequencing for diagnosing complex neurocutaneous syndromes, which helps in guiding clinical management and genetic counseling, especially in consanguineous families. This case expands the known genotypic and phenotypic spectrum of neuro‐ichthyotic disorders and emphasizes the need to consider multilocus pathogenicity in patients with overlapping neurological and dermatological symptoms. Given the severity of the multisystem involvement, seizure burden, and neurodevelopmental impairment, the prognosis remains poor.

## 6 Discussion

Our case represents an unprecedented multilocus genetic etiology for neuro‐ichthyosis. The phenotype is primarily driven by CC2D2A‐related ciliopathy, with ABCA12, DOCK6 variants, and the 14q31.3–q32.11 deletion acting as likely genetic modifiers. While each of these variants has been individually described in the literature, their co‐occurrence in a single patient has not been previously reported. Previous studies have described single‐gene causes of neuro‐ichthyosis [5, 6, 7].

Compared to these reports, our patient exhibits a synergistic phenotype, where multiple variants likely interact to exacerbate disease severity. Unlike classic Joubert syndrome, this patient had profound developmental regression and ichthyosis, suggesting modifying effects from ABCA12, DOCK6, and the 14q deletion.

The genetic findings in this patient are multifaceted, but the primary diagnosis is rooted in the homozygous missense variant in the CC2D2A gene (c.1268G>A, p.Arg423Gln), linking the patient to Joubert syndrome type 9 (JBTS9). We acknowledge the literature regarding conflicting classifications of pathogenicity for this specific variant (as noted in ClinVar, accession: 1058499). However, in the context of the homozygous state in a consanguineous patient with a severe neurological phenotype, this variant is the most plausible primary driver. CC2D2A is known to be a causative gene for approximately 10% of Joubert syndrome cases and encodes a protein crucial for primary cilia formation and function, particularly in Rab8‐dependent vesicle trafficking [9, 10].

The patient's presentation of ichthyosis is supported by a heterozygous variant of uncertain significance (VUS) in the ABCA12 gene (c.5779‐1G>A). The ABCA12 gene is associated with ARCI, a group of disorders characterized by dry, scaly skin [5, 11].

We emphasize that ABCA12‐related ARCI typically requires biallelic mutations for full phenotypic expression. Therefore, the single heterozygous variant in this patient is interpreted as a likely genetic modifier that potentially exacerbates the ichthyotic symptoms, rather than constituting a second independent ARCI diagnosis [5, 11].

Furthermore, a heterozygous likely pathogenic variant in the DOCK6 gene (c.2520dupT) was detected, a gene associated with Adams‐Oliver syndrome type 2 (AOS2). Like ABCA12‐related ARCI, DOCK6‐related AOS2 is typically an autosomal recessive condition. Given the single heterozygous variant, DOCK6 is also considered a potential modifier of the neurodevelopmental and potential vascular phenotype in this patient. DOCK6 functions as a guanine nucleotide exchange factor (GEF) for Rho GTPases, playing a role in cytoskeletal remodeling and neural protrusion growth [5, 12, 13]. Loss‐of‐function mutations in DOCK6 can disrupt actin cytoskeleton organization and lead to abnormalities in embryonic development.

While a heterozygous interstitial deletion of approximately 1 Mbp in the 14q31.3–q32.11 region was found, this deletion encompasses several critical neurodevelopmental genes, notably FOXG1 and BCL11B. Deletions encompassing these genes have been linked to a phenotype including epilepsy, motor and cognitive delay, and speech problems, phenotypes that strongly align with this patient's severe clinical presentation [5, 14].

The clinical and genetic findings in this case significantly expand the known genotypic and phenotypic spectrum of neuro‐ichthyotic syndromes.

Neuro‐ichthyotic disorders are defined by the coexistence of neurological symptoms and ichthyosis, a heterogeneous group of conditions in and of itself [1, 5]. While specific syndromes like Sjögren‐Larsson syndrome (SLS) and multiple sulfatase deficiency (MSD) are well‐established as neuro‐ichthyotic disorders, this case highlights an unprecedented multilocus etiology [1, 5].

The patient's symptoms, driven by the combination of a ciliopathy (Joubert syndrome) and several genetic modifiers, provide evidence for a complex neuro‐ichthyotic phenotype not previously cataloged.

The complex clinical picture of this case emphasizes the need for an integrated diagnostic approach, particularly when a patient presents with severe and drug‐resistant seizures, developmental regression, and ichthyosis. The failure of multiple antiepileptic drugs to provide lasting seizure control highlights the necessity of identifying the underlying genetic cause for a more targeted therapeutic strategy. Early application of Whole Exome Sequencing (WES) is crucial to prevent diagnostic delays, inform prognosis, and enable proper genetic counseling, especially in cases with a family history of consanguinity and similar symptoms. Ultimately, managing the multisystemic challenges of such patients requires the collaborative expertise of a multidisciplinary team [15].

The complex genetic profile of this case presents a compelling opportunity for future research into gene–gene interactions and their synergistic effects. The case highlights the need to create databases and registries specifically for tracking complex multilocus genetic cases, which are often overlooked in large‐scale studies focused on single‐gene disorders. Furthermore, functional studies using model systems are crucial to understand how the combination of variants in CC2D2A, ABCA12, and DOCK6, along with the 14q deletion, affects cellular pathways and development. The unexpected absence of classic Joubert syndrome‐associated neuroimaging findings in this patient also underscores the importance of further research into how genetic modifiers can alter or suppress the typical phenotypic expression of a primary pathogenic variant, ultimately leading to a more nuanced understanding of complex syndromic disorders [5, 9].

## Author Contributions

**Bessan Hamed Dababseh:** project administration, writing – original draft. **Ala'a S. Ghnimat:** project administration, writing – original draft. **Lubna W. AbuHamdiya:** project administration, writing – original draft. **Mones H. Atatre:** project administration, writing – original draft. **Ahmad Batran:** project administration, supervision. **Mahdi W. Suboh:** conceptualization, project administration, supervision, writing – original draft, writing – review and editing.

## Funding

The authors have nothing to report.

## Consent

Written informed consent was obtained from the patient's parents for the publication of this case report.

## Conflicts of Interest

The authors declare no conflicts of interest.

## Data Availability

The data used to support the findings of this study are included in the article.
